# Organizational approaches to collaboration in vocational rehabilitation—an international literature review

**DOI:** 10.5334/ijic.670

**Published:** 2011-11-18

**Authors:** Johanna Andersson, Bengt Ahgren, Susanna Bihari Axelsson, Andrea Eriksson, Runo Axelsson

**Affiliations:** Nordic School of Public Health, Gothenburg, Sweden; Nordic School of Public Health, Gothenburg, Sweden; Nordic School of Public Health, Gothenburg, Sweden; Nordic School of Public Health, Gothenburg, Sweden; Sahlgrenska Academy, University of Gothenburg, Sweden and Aalesund University College, Norway

**Keywords:** interorganizational relations, collaboration, vocational rehabilitation, organizational models, barriers, facilitators

## Abstract

**Introduction:**

Collaboration between welfare organizations is an important strategy for integrating different health and welfare services. This article reports a review of the international literature on vocational rehabilitation, focusing on different organizational models of collaboration as well as different barriers and facilitating factors.

**Methods:**

The review was based on an extensive search in scientific journals from 1995 to 2010, which generated more than 13,000 articles. The number of articles was reduced in different steps through a group procedure based on the abstracts. Finally, 205 articles were read in full text and 62 were included for content analysis.

**Results:**

Seven basic models of collaboration were identified in the literature. They had different degrees of complexity, intensity and formalization. They could also be combined in different ways. Several barriers and facilitators of collaboration were also identified. Most of these were related to factors as communication, trust and commitment.

**Conclusion:**

There is no optimal model of collaboration to be applied everywhere, but one model could be more appropriate than others in a certain context. More research is needed to compare different models and to see whether they are applicable also in other fields of collaboration inside or outside the welfare system.

## Introduction

Integration has become an important issue in the development of the modern welfare society. Different health and welfare services have become more and more specialized and they are provided by an increasing number of different organizations, not only government agencies but also non-governmental organizations, community groups and private enterprises. This differentiation of providers has generated a corresponding need for integration in order to avoid a fragmentation of services [1].

Collaboration between welfare organizations is a strategy to integrate the different health and welfare services [2]. During the past 20 years there have been many initiatives to improve the collaboration between organizations in different parts of the welfare system, for example in vocational rehabilitation, care of the elderly and the functionally disabled, open psychiatric care and other forms of community care [3–6].

Vocational rehabilitation is a multidisciplinary intervention to help individuals to return to work after an occupational injury, or a period of unemployment or sickness, i.e., labor market integration or reintegration. This involves actors from many different professions, organizations and sectors of the society [7, 8]. Depending on the type of welfare system, vocational rehabilitation usually includes different health and social services, occupational health services, employment services, and social or private insurance. In addition, employers and trade unions are often involved as well as the individuals concerned.

With all of these different actors, there is an obvious risk of service fragmentation in vocational rehabilitation. Individuals may fall between the stools of the different professions and organizations involved, or there may be a costly duplication of services [2]. In order to avoid such a fragmentation, there have been initiatives to improve collaboration between organizations involved in vocational rehabilitation. In Sweden, for example, there have been a number of experiments with different models of collaboration. In this connection, a number of barriers as well as facilitating factors have also been observed [9].

Against this background, the purpose of the article is to present a review of the international literature reporting research on collaboration in vocational rehabilitation. The review focused on identifying, describing and comparing different organizational models of collaboration as well as different barriers and facilitators.

## Materials and methods

This review was made by a research group at the Nordic School of Public Health. The group consists of five members (the authors) with different educational and professional backgrounds in health management, human resource management and public health. The review was based on an extensive search for literature on collaboration in vocational rehabilitation. The search was limited to articles in peer reviewed scientific journals from 1995 and later. It was also limited to journals in English language. The search was made in MEDLINE, Cinahl, ISI and parts of CSA (ASSIA, PAIS International, PsycARTICLES, PsycINFO, Social Services Abstracts, Sociological Abstracts and Worldwide Political Science Abstracts). These databases were chosen in order to have a broad selection of perspectives on collaboration in vocational rehabilitation. The search was made in April 2010.

A wide range of terms was used in the literature search. There is a conceptual confusion regarding integration and collaboration, which has often been pointed out in the literature [10, 11]. Moreover, there are different concepts used in connection with vocational rehabilitation. Therefore, it was necessary to use a number of different search terms like vocational or occupational rehabilitation, return to work, collaboration, cooperation, coordination, cooperative behavior, partnership, inter- or multidisciplinary, interprofessional, interorganizational and intersectoral.

The search generated a total of 13,132 articles, but an initial review of their titles reduced the number of articles to 1005. Articles were excluded at this stage if the titles showed that they were clearly outside the scope of the study, for example collaboration in paediatrics, geriatrics and palliative care, or in rehabilitation of ecosystems. The abstracts of the remaining 1005 articles were distributed within the research group and read by two members of the group independent of each other. Abstracts of articles by someone from the research group were read and reviewed by other members of the group.

An article was included for further review if it was an empirical, peer reviewed study concerning both collaboration and vocational rehabilitation. Different research designs were included, such as case studies, qualitative interview studies and quantitative studies using questionnaires. Theoretical articles or articles suggesting collaboration as a solution to a perceived problem were excluded as well as studies of interprofessional education. Furthermore, in order to be included, the collaboration studied had to be between different professionals or organisations, for example not just between doctors and nurses as part of their regular work. Articles focusing only on the effects of vocational rehabilitation and not describing any models of collaboration were also excluded.

If agreement on inclusion or exclusion of an article could not be reached between those who had read the abstract, the whole group discussed it until consensus was reached. This procedure reduced the number of abstracts to 205, which were read in full text according to the same procedure. Finally, 62 articles were included for closer analysis. These articles were read and summarized in a protocol extracting relevant information such as aim, design, data collection, analysis, description of collaboration model and target group, conclusions and what could be learnt in terms of barriers or facilitators. This extraction of information was very close to the original articles.

The information extracted from the articles was analyzed using qualitative content analysis [12]. It was made by first reading the extracted material and then sorting it into categories of models, barriers and facilitators of collaboration in vocational rehabilitation. The analysis was guided mainly by considerations of difference and frequency. The resulting categories were then labeled according to their contents. The labels on the different models are somewhat abstracted, which means that they are not always coinciding with the terminology used in the original articles. The labels on the different barriers and facilitators are closer to the terminology of the articles.

## Results

Many of the studies reviewed came from Sweden, which is not so surprising considering the extensive experiments with collaboration in vocational rehabilitation that have taken place in this country during the last fifteen years [9]. Other countries represented were Canada, USA, the Netherlands, UK, Australia, Belgium and Norway. There were studies based on quantitative as well as qualitative data. From these studies a number of different models of collaboration were derived, and also a number of different barriers and facilitating factors.

### Models of collaboration

The review of the literature shows that there are different organizational models of collaboration used in connection with vocational rehabilitation. These models have been developed in different welfare systems, with different actors and different target groups. As a result, the models have different degrees of complexity, intensity and formalization [13]. Many of them are also used in combination. Even so, there seems to be a limited number of basic organizational models of collaboration in vocational rehabilitation. In the analysis of the articles reviewed, the following seven models were identified.

**Information exchange** between different organizations involved in vocational rehabilitation is the simplest model of collaboration. It is often based on informal contacts between professionals in the different organizations who are working with the same client or patient [14], but it could also be formalized into more systematic consultations between the different professionals involved [15, 16]. The exchange of information can be either verbal or in writing, and it can be supported by information and communication technology such as computer platforms or video conferences [17, 18].

**Case coordination** is a model where the different organizations involved are not collaborating directly with each other, but indirectly through a person who is coordinating their different rehabilitation activities towards an individual [19, 20]. A case coordinator is often employed by one of the organizations, but works as a personal agent for the client or patient, guiding him or her through the whole rehabilitation process. In this work, the case manager is balancing the activities of the different organizations, trying to mediate a common understanding and a common plan for rehabilitation of the individual concerned [21, 22].

**Interagency meetings** are often arranged in connection with vocational rehabilitation. This is a model of collaboration where professionals from the different organizations involved meet to discuss individual clients or patients that they have in common, sometimes together with the individual concerned. The aim of these meetings is to agree on common activities in the rehabilitation process [23–26]. The meetings may be more or less systematic, following formal procedures for planning and implementation of different rehabilitation activities [27, 28]. Some of these procedures are also claimed to be more or less evidence based [29, 30].

**Multidisciplinary teams** are used both as a working mode and a model of collaboration in vocational rehabilitation. In this model, a group of professionals from different organizations are working together continuously and over a longer period as a team for rehabilitation of individual clients or patients. The different professionals have complementary competences and they bring their expertise to the team [31–34]. There are many different teams in vocational rehabilitation, for example clinical teams and intervention teams [35]. These teams may be multiprofessional or interprofessional and sometimes even transprofessional, depending on the intensity of the contacts between the members and how dependent they are on each other [36].

**Partnership** is a model of collaboration, which is used in many different contexts and also in vocational rehabilitation. There are different forms of partnership, but it is always based on formal agreements between two or more organizations to integrate their services across organizational boundaries. In vocational rehabilitation there may be formal agreements on collaboration between the different organizations involved [37, 38], or formal structures may be established for communication and exchange of information between these organizations [39]. Such agreements can include different responsibilities and obligations for the organizations involved and they can be more or less formalized, although partnership always means formalization to some extent [40].

**Co-location** is not so much an organizational model of collaboration, but rather a model creating favorable conditions for interorganizational collaboration. In this model, different organizations involved in vocational rehabilitation, or parts of these organizations, are located in the same premises [30, 41]. This means a physical proximity, which may have positive effects on the contacts and the communication between the professionals of the different organizations [42]. At the same time, it may serve as a common entrance or reception for the clients or patients to the different organizations and rehabilitation services [43].

**Pooling of budgets** is the most complex and demanding model of collaboration in vocational rehabilitation. In addition to a close collaboration between the organizations and professionals involved, a joint budget is created by pooling some financial resources from the different organizations. The pooled budget is a result of negotiations between the organizations and is underpinned with legal arrangements. It is used to finance joint rehabilitation activities or projects, which are planned in collaboration between the organizations involved [44–47]. Sometimes the organizations are also forming a separate structure for collaboration, within which different rehabilitation activities can take place [48, 49].

**Figure 1.**
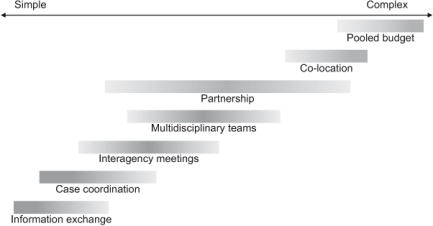
Models of collaboration identified in the literature review.

The different models of collaboration are summarized in Figure 1, where the complexity of the models and their relationship to each other as well as their variations is indicated by the length and darkness of the lines.

As mentioned before, these models are not exclusive and they often appear in combination. For example, information exchange can be combined with most of the other models. In the same way, a case coordinator is often combined with interagency meetings or a multidisciplinary team [26, 50]. Partnerships may also be combined with co-location as well as pooling of budgets [34, 42].

### Barriers and facilitators

Research on interprofessional and interorganizational collaboration has been focusing a great deal on problems and difficulties in collaboration [51]. The same goes for research on collaboration in vocational rehabilitation. There are a number of barriers to collaboration described in the articles reviewed, but there are also a number of facilitating factors. The barriers and the facilitators are often described in the same terms and many of them seem to be two sides of the same coin.

Many of the barriers to and facilitators of collaboration in vocational rehabilitation are related to the *communication* between the organizations and professionals involved [21, 33, 52]. A lack of communication or an insufficient dialogue between the different actors is a barrier [53], which may lead to ambiguity regarding the different roles and responsibilities in the rehabilitation process [14, 26, 37]. In the absence of communication, the actors may also have different views on the aims and goals of the collaboration [40, 45]. On the other hand, if the different actors are communicating with each other, they can increase their knowledge of each other [54, 55] and develop a mutual understanding and respect [19], which may facilitate collaboration.

The *trust* between the organizations and professionals involved in collaboration is another important factor, which is regarded mainly as a facilitator of collaboration [49, 53]. Trust may be a result of communication between the different actors, but also of a long-term commitment to collaboration [56]. According to the literature, it takes a long time to build trust, but it can be destroyed in a very short time. It is therefore important to have enough time to build trust between the different actors involved in collaboration [24]. It is also important to have a continuity of actors over a longer period [28, 57].

A lack of trust between the different actors involved in vocational rehabilitation may lead to suspicion and territorial behavior. There are also a number of barriers to collaboration related to *territoriality*. If the different organizations involved are focusing on their own interests, what they can get out of the collaboration, there may be territorial conflicts between the organizations [39, 58]. There could also be competition for power and resources between the professionals involved [33]. These barriers may lead to a fragmentation between the collaborating actors, which may seriously impair collaboration.

The opposite of territoriality is when the different organizations and their professionals are able to establish a *common ground* for collaboration [28, 37]. There are a number of facilitators related to the existence of a common ground among the actors involved in vocational rehabilitation. It is built on trust between the different actors and a focus on the needs of their common clients or patients. In addition, it may include shared aims and goals [45], a common language for dealing with vocational rehabilitation [34, 40, 43], and a common culture of collaboration. These things take time to develop, but they may be supported by joint training in interprofessional collaboration [24, 28].

There are a number of barriers and facilitators related to the *commitment* of the actors involved in collaboration [33, 56]. Their commitment is based on communication, trust and a common ground for collaboration, but also on other conditions for collaboration. One important condition is the target group for vocational rehabilitation. It may be a facilitator if it is clearly defined [34], but a barrier if there are different views on the constitution of the group [43, 46]. Another condition is the involvement of actors in the collaboration. It may be a facilitator if all the relevant actors are involved [21, 52, 59], but a barrier if important actors are missing [14, 24]. In connection with involving all relevant actors, the interdependence of the different actors is particularly important [31, 38].

*Rules and regulations* are regarded mainly as barriers to collaboration in vocational rehabilitation. For example, there may be different rules on confidentiality and employment conditions in the organizations involved, which are complicating the collaboration between professionals [25, 44]. These rules and regulations may change over time in the different organizations, due to new legislation or political initiatives, which may create new barriers to collaboration [33, 57, 60]. On the other hand, rules and regulations in terms of formal procedures and systematic planning can also be regarded as facilitators [25, 28].

There are both barriers and facilitators of collaboration related to *leadership*. A leader who is defending the territory of his or her organization is a barrier to collaboration, while a leader who is able to transcend organizational boundaries may facilitate collaboration. The opposite of a territorial leader is an altruistic leader, who is prepared to give up territory in order to achieve a better total outcome for the clients or patients concerned [58]. A leader may give support to the professionals involved in collaboration, which is an important facilitator [38, 43]. Such support can be in the form of time [30, 33] or resources for collaboration [38], but it may also be in the form of a mandate for the professionals to represent their organization [24]. In any case, a supportive leadership also has to be adjusted to the model of collaboration [36].

## Discussion

Seven basic models of collaboration in vocational rehabilitation have been identified in this review: information exchange, case coordination, interagency meetings, multidisciplinary teams, partnership, co-location, and pooling of budgets. As mentioned before, these organizational models have different degrees of complexity, intensity and formalization. The models were also presented along an implicit scale of complexity, as shown in Figure 1, starting with the simple model of information exchange and ending with pooling of budgets, which was described as the most complex and demanding model.

These models of collaboration could also be placed along a continuum of integration with different degrees of intensity or formalization of connections between the organizations involved [13, 61]. On such a continuum, the different models would be placed in a similar order, from information exchange as the most loosely coupled and informal model of collaboration to pooling of budgets as the most intensive and formalized. It is important to point out, however, that the optimal degree of intensity or formalization in collaboration varies, and so does also the optimal degree of complexity, since the need for integration is dependent on the degree of differentiation or fragmentation [2, 62].

The different models of collaboration could be presented in another order if they were classified in terms of the organizational levels where they are located or their targets [63]. Information exchange, case coordination, interagency meetings and multidisciplinary teams are located mainly on the organizational micro level of service production, while the other models are including middle management levels as well. Even the top management level is involved in partnerships and pooling of budgets. The models on the micro level are organized around an individual client or patient, while the other models are focusing more on populations or larger target groups.

The different models could also be classified as structural or process oriented [64, 65]. Case coordination, partnership, co-location and pooling of budgets are mainly structural models, while information exchange, interagency meetings and multidisciplinary teams are more process oriented. In the same way, the structural models could be classified as interorganizational and the process oriented models as interprofessional. All of these classifications are however largely overlapping [1].

The different models of collaboration are used not only in vocational rehabilitation, but also in many other parts of the welfare system. For example, case coordination has been used for a long time in psychiatric care [66] and partnerships are often used in care of the elderly and different forms of community care [6]. Pooling of budgets has been developed into a structure or an arena for collaboration in the Swedish experiments in vocational rehabilitation, but a similar organizational model is used also in the UK for collaboration between health and social care [44].

It has often been pointed out in the literature that there is not an optimal model of collaboration that can be applied everywhere, but one model may be more appropriate than others in a certain context [2, 67]. It is therefore important to discover and recognize the ‘collaborative advantage’ of the different organizational models [51]. Vocational rehabilitation includes processes such as return to work, labor market integration and reintegration, which require different models of collaboration. Different target groups need different models and the same target group may need different models in different phases of rehabilitation [68]. It is therefore important to have a flexible approach to collaboration and a repertoire of different organizational approaches.

Several barriers and facilitators of collaboration in vocational rehabilitation have been identified in this literature review. The barriers are often described in the same terms as the facilitators and many of them seem to be two sides of the same coin, for example those related to communication, commitment, and leadership. The other barriers and facilitators are more one-sided, for example those related to trust or territoriality, but it seems that they are also working both ways. Because of their close relationships, the different barriers and facilitating factors may be grouped together into a smaller number of *determinants* working both as barriers and facilitators, which have also been indicated, in italics, in the presentation [69].

The determinants of collaboration may sometimes overlap in a hypothetical chain. For example, improved communication between the actors involved in vocational rehabilitation can increase their knowledge of each other and lead to mutual understanding and respect, which may also improve trust between them. Because of this overlap it is more difficult to sort out the different determinants than the different models of collaboration, although it might be possible to relate them in a similar way to different organizational levels, structural and process oriented factors, or interorganizational and interprofessional collaboration. The determinants could also prove to be more or less important to collaboration in a short- or long-term perspective. A collaboration intended to last for a long time may need other ways to overcome barriers than one with a short duration.

In the same way as the barriers and facilitators may be regarded as determinants of collaboration, the different models may also be regarded as *strategies* for collaboration, which may be combined in different ways depending on the collaborative advantage of different combinations. As mentioned before, the different organizational models of collaboration often appear in combinations. Together they may even create new hybrid models, like case coordinators supported by interagency meetings, or multidisciplinary teams supported by co-location or pooling of budgets. However, a pooled budget on the macro level may facilitate but cannot alone create integrated health and welfare services. It has to be combined with collaboration between professionals on the micro level of the organizations involved [70].

Most of the articles in this literature review were studies of collaboration in vocational rehabilitation from a professional or organizational point of view. There were few studies taking the views of the service users into consideration [48]. This may be explained by the aim of this review, which was to identify different models, barriers and facilitating factors in collaboration and not the possible effects of vocational rehabilitation, where it might have been more relevant to study the experiences of the service users. Furthermore, there are some studies reporting that service users may not be aware of existing collaboration [71, 72].

## Conclusions

In this review seven basic models of collaboration in vocational rehabilitation have been identified and described. These organizational models have different degrees of complexity, intensity and formalization, but there is not one optimal model of collaboration in vocational rehabilitation that can be applied everywhere. On the other hand, one model may be more appropriate than others in a certain context, depending most of all on the needs of the clients or patients concerned.

Barriers and facilitators of collaboration are often described as two sides of the same coin. There are a great number of different barriers and facilitating factors, but because of their close relationships they may be grouped together into a smaller number of determinants working both as barriers and facilitators. In the same way, the different models of collaboration may be regarded as strategies for collaboration, which can be combined in different ways depending on the collaborative advantage of different combinations.

The models of collaboration described in this article are not unique for vocational rehabilitation. Similar models are used also in care of the elderly and the functionally disabled, open psychiatric care and other forms of community care. This means that experiences from these fields may be useful for vocational rehabilitation. On the other hand, the experiences of different models, barriers and facilitators of collaboration in vocational rehabilitation may be useful for other parts of the welfare system and also for collaboration between organizations and professionals outside the welfare system. The increasing differentiation of services and providers is a general feature of the modern society, which requires a corresponding integration in order to avoid fragmentation.

More research is needed to explore to what extent the results of this review are applicable in and transferable to other fields inside or outside the welfare system. There is also a need for comparative studies of the different models of collaboration, their specific barriers and facilitators, and whether some models are more commonly used than others. The determinants as well as the strategies for collaboration have to be further explored. Moreover, since most of the studies reviewed have focused on collaboration in vocational rehabilitation from a professional or an organizational point of view, there is a need for studies focusing on the experiences of the clients or patients as well.

## Reviewers

**Bjorn Jakobsson**, PhD, Department of Health Sciences, Mid Sweden University, Campus Ostersund, SE- 831 25 Ostersund, Sweden

**William S. Shaw**, PhD, Principal Research Scientist, Liberty Mutual Research Institute for Safety, Hopkinton, MA, USA

**Lasse Skovgaard**, MA, MA (Ed.), PhD-fellow, The Danish Multiple Sclerosis Society, Mosedalvej 15, 2500 Valby, Denmark; Department of Public Health, University of Copenhagen, Øster Farimagsgade 5, 1014 Copenhagen K, Denmark; National Research Center in Complementary and Alternative Medicine, NAFKAM, Faculty of Health Science, University of Tromsø, N – 9037 Tromsø, Norway
